# Effects of UV Treatment on Ceria-Stabilized Zirconia/Alumina Nanocomposite (NANOZR)

**DOI:** 10.3390/ma13122772

**Published:** 2020-06-18

**Authors:** Satoshi Komasa, Seiji Takao, Yuanyuan Yang, Yuhao Zeng, Min Li, Sifan Yan, Honghao Zhang, Chisato Komasa, Yasuyuki Kobayashi, Hiroshi Nishizaki, Hisataka Nishida, Tetsuji Kusumoto, Joji Okazaki

**Affiliations:** 1 Department of Removable Prosthodontics and Occlusion, Osaka Dental University, 8-1 Kuzuha-hanazono-cho, Hirakata, Osaka 573-1121, Japan; komasa-s@cc.osaka-dent.ac.jp (S.K.); takao-s@cc.osaka-dent.ac.jp (S.T.); yang-y@cc.osaka-dent.ac.jp (Y.Y.); zeng-y@cc.osaka-dent.ac.jp (Y.Z.); liminmin0529@gmai.com (M.L.); yan-z@cc.osaka-dent.ac.jp (S.Y.); zhang-h@cc.osaka-dent.ac.jp (H.Z.); terada-c@cc.osaka-dent.ac.jp (C.K.); 2 Osaka Research Institute of Industrial Science and Technology, Morinomiya Center, 1-6-50, Morinomiya, Joto-ku, Osaka 536-8553, Japan; kobaya@omtri.or.jp; 3 Department of Japan, Faculty of Health Sciences, Osaka Dental University, 1-4-4, Makino-honmachi, Hirakata-shi, Osaka 573-1121, Japan; nisizaki@cc.osaka-dent.ac.jp (H.N.); kusumoto@cc.osaka-dent.ac.jp (T.K.); 4 Department of Advanced Hard Materials, The Institute of Scientific and Industrial Research (ISIR), Osaka University, Osaka 567-0047, Japan; hnishida@sanken.osaka-u.ac.jp

**Keywords:** NANOZR, UV treatment, implant, bone differentiation, angiogenesis, periodontal tissue regeneration

## Abstract

Nanostructured zirconia/alumina composite (NANOZR) has been explored as a suitable material for fabricating implants for patients with metal allergy. In this study, we examined the effect of UV treatment on the NANOZR surface. The experimental group was UV-treated NANOZR and the control group was untreated NANOZR. Observation of the surface of the UV-treated materials revealed no mechanical or structural change; however, the carbon content on the material surface was reduced, and the material surface displayed superhydrophilicity. Further, the effects of the UV-induced superhydrophilic properties of NANOZR plates on the adhesion behavior of various cells were investigated. Treatment of the NANOZR surface was found to facilitate protein adsorption onto it. An in vitro evaluation using rat bone marrow cells, human vascular endothelial cells, and rat periodontal ligament cells revealed high levels of adhesion in the experimental group. In addition, it was clarified that the NANOZR surface forms active oxygen and suppresses the generation of oxidative stress. Overall, the study results suggested that UV-treated NANOZR is useful as a new ceramic implant material.

## 1. Introduction

The use of metal implants as prosthetics has become an essential treatment for tooth defects [1,2]. Successful initial fixation of the implant depends on osseointegration, the direct deposition of the bone on the surface of an implant without the intervention of soft/connective tissue [3,4,5,6,7,8]. Zirconia is a new biomaterial that has garnered much attention. It is being widely used, after titanium, especially in dentistry [9,10,11,12]. Zirconia was introduced 20 years ago and solved the problem of alumina brittleness and the associated risk of implant failure. Zirconium exists in one of three crystalline forms: monoclinic, tetragonal, and cubic. To obtain mechanical stability at room temperature, commercially available zirconia is mixed with other metal oxides, such as MgO, CaO, and Ce_2_O_3_.

Nawa et al. combined ceria-stabilized tetragonal zirconia polycrystal (Ce-TZP) and alumina polycrystal (Al_2_O_3_) to create a nanostructured zirconia/alumina composite (NANOZR) based on a ceria-stabilized tetragonal ZrO_2_ polycrystal ceramic [13,14,15]. This material is considered to be useful for dental implants as a material that undergoes low temperature deterioration. NANOZR has higher flexural strength and fracture toughness than 3 mol% yttria-stabilized tetragonal zirconia polycrystal (3Y-TZP) [16,17]. NANOZR has been applied in various fields due to its properties and is expected to be applied not only as a denture base material but also as an implant material. However, since zirconia is biologically inert, it is necessary to perform surface modification to allow the living tissue in contact with the material to establish favorable interactions with it. It has been shown in past reports that NANOZR is a material capable of inducing hard tissue differentiation [13,14].

In recent years, implants with various surface shapes and properties have been used to promote bone formation at the interface between the dental implant and the bone and to achieve osseointegration at an early stage [18,19,20,21,22]. Various studies have been conducted on what kind of design should be introduced on the surface of the implant material; however, there is no clarity on the kind of surface modifications necessary [23,24,25]. Physical methods of surface treatment of implant materials include ultraviolet treatment and low-temperature plasma treatment, and chemical methods include blast treatment, acid etching treatment, hydrogen peroxide solution treatment, and sodium hydroxide treatment [26,27]. It is presumed that these surface treatments are effective for implant materials because they mechanically change the surface of the material, increase the cell contact area, and remove carbon from the material surface and make it hydrophilic [28,29,30]. Our research team has been studying the surface modification of implant materials. An alkali-modified titanium surface exhibits smooth nanometer-scale surface roughness [31]. It has been clarified both in vitro and in vivo that pure titanium metal with nanostructure improves biocompatibility [32,33,34]. In addition, it has been clarified that this material is useful not only for hard tissue formation around the implant placement tissue but also for regeneration of periodontal tissue after angiogenesis and wound healing [35,36].

It has also been shown that TNS can easily adsorb various proteins due to its chemical properties. In our previous report, we showed that this material surface could promote the adsorption of amelogenin, which helped regenerate hard and periodontal tissues [37]. Moreover, we have been exploring the use of UV treatment and atmospheric-pressure plasma treatment to modify the surfaces of various materials [38,39]. Since these treatments can be carried out relatively easily, active oxygen species on the surfaces of materials can be reduced, and the surfaces can be rendered superhydrophilic, making them well-suited for facilitating biofunctionalization of implants. While several reports have demonstrated the potential applications of these treatments for titanium surfaces [38,39], these treatments are necessary to impart biofunctionalization on zirconia surfaces to utilize the material for fabricating superior implants and for other biomedical applications.

Previously, we had shown that alkali treatment of NANOZR accelerates osseointegration both in vitro and in vivo [40,41]. By surface-treating NANOZR, it was clarified that the initial adhesion of rat bone marrow cells and the ability to induce hard tissue differentiation were improved, and it was revealed that the data are almost the same as those of titanium surfaces [42]. However, the ability of alkali-treated titanium to induce hard tissue differentiation was better, although the surface of this material is hydrophilized. Our previous report showed that UV treatment of titanium surface resulted in high hard tissue differentiation inducing ability and antibacterial properties [38]. However, it was not clear what kind of change occurred on the material surface. Therefore, the purpose of this research was to examine the kind of changes that occur on the surface of the NANOZR material upon UV treatment. In addition, we report the in vitro level analysis of the effects of NANOZR on surrounding tissues after implantation.

## 2. Materials and Methods

### 2.1. Sample Preparation

NANOZR discs (15 mm diameter and 1.5 mm thickness, Yamamoto Kinzoku, Osaka, Japan) were used in the present study. NANOZR discs were morphologically modified by Sarton Works (Yokohama, Japan) and then polished with diamond particle slurry paste (Compact Desktop Wrapping System EJ-3801N, ENGISS JAPAN, Yokohama, Japan). After polishing, NANOZR disks were ultrasonically rinsed in acetone, ethanol, and distilled water for 10 min each and then air-dried. Half of the NANOZR disks were modified with UV light (183 nm, 8 mW/cm^2^, Incident angle to material surface is 90°) for 12 min (TheraBeam^®^ Super Osseo, Ushio Inc., Tokyo, Japan). Samples that were not exposed to UV radiation were used as controls.

### 2.2. Characterization of Materials

Scanning electron microscopy (SEM) (S-400; Shimadzu, Kyoto, Japan) and scanning probe microscopy (SPM) (SPM-9600; Shimadzu, Kyoto, Japan) were used for comparatively evaluating the surface properties of UV-treated and untreated NANOZR disks. X-ray photoelectron spectroscopy (XPS) (Kratos Analytical Axis Ultra DLD electron spectrometer; Kratos Instruments, Manchester, UK) with a monochromatic Al Kα X-ray source was used for analyzing the components of samples after argon-ion etching for 2 min (evaporation rate 5 nm/min). This procedure was performed to remove surface contaminants. Sample surface wettability were analyzed using a contact angle measurement system (VSA 2500 XE, AST Products, Tokyo, Japan). The material used was ultrapure water.

### 2.3. Protein Adsorption

Pierce^TM^ BCA Protein Assay Reagent Kit (Pierce Biotechnology) was used by evaluating protein adsorption on NANOZR disks of test and control group. Bovine serum albumin (BSA) fraction V solution (1 mg/mL protein in saline; Pierce Biotechnology, Rockford, IL, USA) was used for samples. A volume of 300 µL of was placed onto both test and control discs. The evaluation time was 1 h, 3 h, 6 h, and 24 h after dropping BSA on samples. After the lapse of various times, BSA not adhered to the NANOZR surface was recovered. The amount of adhered BSA was measured from the amount of BSA not adhered.

### 2.4. Cell Culture

Animal experimentation in this study was performed under the Guideline for Animal Experimentation at Osaka Dental University (approval no. 19-06001). Rat bone marrow cells (RBMCs) were obtained from the femurs of eight-week-old Sprague-Dawley rats (SHIMIZU Laboratory Supplies Co., Kyoto, Japan). Hind limb bones of rats were aseptically excised after euthanizing rats with 4% isoflurane. The method of extraction and primary culture of bone marrow mesenchymal cells from rat femur is in accordance with our previous reports [32,33,34,35,36,37,38,39,40,41]. RBMs were cultured in 75-cm^2^ culture flasks (BD Biosciences, Franklin Lakes, NJ, USA).

Human umbilical vein endothelial cells (HUVECs) were purchased from CellWorks (Buckingham, UK). The culture was performed on a type 1 collagen-coated disc according to the culture method that we have already reported [35].

Rat periodontal ligament cells (RPLCs) were purchased from Lonza (Walkersville, MD, USA). The cells were cultured in a medium (BulletKit™; Lonza, Basal, Switzerland) prepared at an appropriate concentration (1 mL/cm^2^). Regarding the primary culture and subculturing method of the RPLCs, we follow our previous reports [37].

After confirming that RBMCs, HUVECs and RPLCs became confluent, it was seeded at a rate of 4 × 10^4^ cells/cm^2^ on the surface of NANOZR treated with NANOZR and UV. The medium of each cell on the material surface was changed every three days.

### 2.5. Cell Adhesion

RBMCs, HUVECs and RPLCs were seeded onto the specimens at an initial density of 4 × 10^4^ cells/cm^2^ and allowed to attach for 1 h, 3 h, 6 h, and 24 h. The number of RBMCs, HUVECs and RPLCs adhesions was examined by CellTiter-Blue*^®^* Reagent (50 μL CellTiter-Blue*^®^* Reagent diluted in 250 μL PBS). The analysis method follows the manufacturer’s instructions. According to our past report, RBMCs and RPLCs (after 24 h of culture) and HUVECs (after 6 h of culture) on the test and control NANOZR surfaces were stained and observed by confocal laser scanning microscopy [32,33,34,35,36,37,38,39,40,41].

### 2.6. qRT-PCR, Alkaline Phosphatase Activity, DNA Content, and Mineralization Determination

Expression of osteogenesis-related genes was assessed using a real-time TaqMan RT-PCR assay (Life Technologies, Carlsbad, CA, USA). Total RNA was extracted using an RNeasy Mini Kit (Qiagen, Venlo, the Netherlands), and 10-μL aliquots of each RNA sample were reverse transcribed into cDNA utilizing a Prime Script RT Reagent kit (TaKaRa Bio., Shiga, Japan). We investigated alkaline phosphatase (ALP) activity at day 7, runt-related transcription factor (Runx2) at day 3, bone morphogenetic protein 2 (Bmp-2) at day 14, Bglap at day 21, ICAM-1 at day 3, von Willebrand factor at day 7, and thrombomodulin mRNA at day 14.

In order to evaluate ALP activity, following seven or 14 days of incubation, samples were washed with PBS, and RBMCs and RPLCs that had attached to the sample surface were dissolved with 300 μL of 0.2% Triton X-100. ALP activity was evaluated by an alkaline phosphatase luminometric enzyme-linked immunosorbent assay (ELISA) kit (Sigma-Aldrich) in accordance with the manufacturer’s instructions. A PicoGreen dsDNA analysis kit (Invitrogen/Life Technologies) was utilized to evaluate the DNA content. The amount of ALP was normalized to the amount of DNA in each cell lysate.

Following 21 or 28 days of incubation, calcium deposition in the extracellular matrix was measured after dissolution with 10% formic acid. Calcium content was quantified and calculated using a Calcium E-test Kit (Wako Pure Chemical Industrials Ltd. Osaka, Japan) according to the manufacturer’s instructions.

### 2.7. Cell Intracellular Reactive Oxygen Species (ROS) Level and Mitochondrial Membrane Potential Change Detection of RBMCs

The intracellular ROS level was determined using CellROX^®^ oxidative stress reagents (C10422, Thermo Fisher Life Technologies Ltd., Tokyo, Japan). The cells were washed with PBS three times and incubated in medium containing 5 μM CellROX^®^ oxidative stress reagents for 30 min at 37 °C. RBMCs were collected by trypsinization and then transferred into a 96-well plate. ROS levels on RBMCs of the test and control NANOZR disks were stained and observed by confocal laser scanning microscopy (LSM700, Zeiss, Oberkochen, Germany).

Mitochondrial membrane potential (ΔψM) is an important parameter for mitochondrial function in understanding the relationship between ROS and RBMCs. The JC-1 kit (10009172, Cayman, Ann Arbor, MI, USA) was used to determine if UV treatment caused changes in mitochondrial membrane potential of RBMCs. The mitochondrial membrane potential of RBMCs at the test and control NANOZR surface was visualized using a confocal laser scanning microscope (LSM700).

### 2.8. Statistical Analyses

All data were expressed as the mean ± standard deviation. Each experiment was repeated four times, and all results were compared in SPSS 26.0 by Student’s *t*-test; *p* < 0.05 was considered statistically significant.

## 3. Results

### 3.1. Evaluation of NANOZR Sample

**Figure 1:**
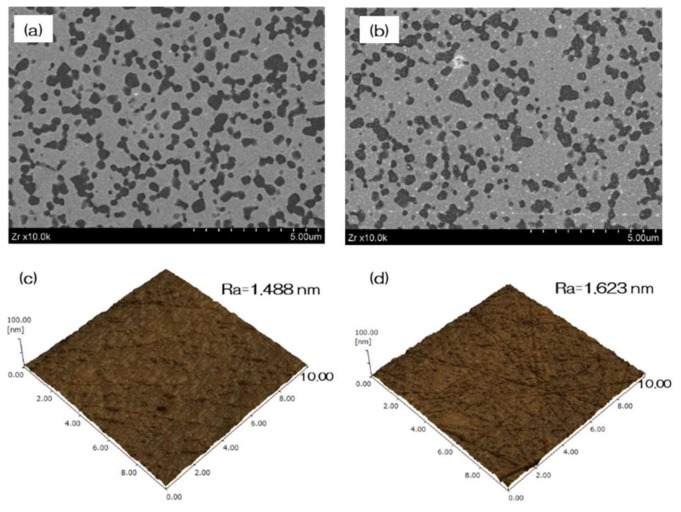
The surface characterization of NANOZR samples. In the scanning electron microscopy (SEM) evaluation, no change was observed on the surface of the NANOZR by UV treatment (**a**—test; **b**—control). Scanning probe microscopy (SPM) analysis also showed no change in the surface roughness of the test disks (**c**—test; **d**—control).

**Figure 2:**
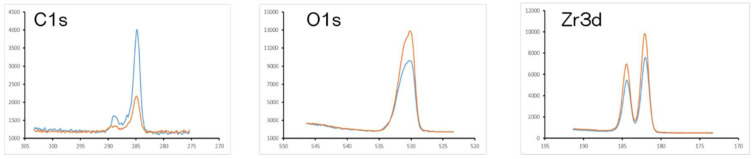
Orange line—test, blue line—control. The X-ray photoelectron spectroscopy (XPS) analysis of the surface UV-treated NANOZR and untreated NANOZR are shown. XPS analysis showed the increase in the O1s peak and decrease in the C1s peak on the surface of UV-treated NANOZR.

**Figure 3:**
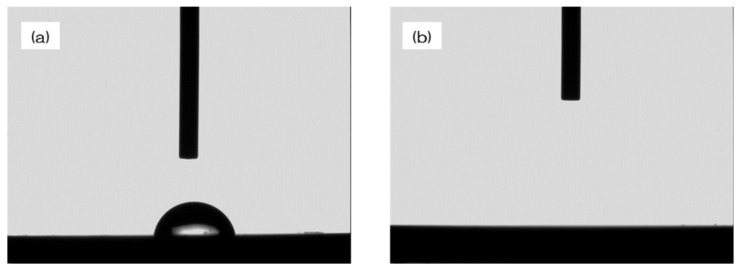
The contact angle between the surface of the NANOZR treated with UV and the surface of the untreated NANOZR is shown. The UV-treated NANOZR surface was superhydrophilic. (**a**—test; **b**—control).

Figure 1 shows the evaluation of NANOZR samples. In the SEM evaluation, no change was observed on the surface of the NANOZR by UV treatment (Figure 1a,b). SPM analysis also showed no change in the surface roughness of the test disks (Figure 1c,d). According to the result of XPS analysis, an increase of O_2_ peak on the surface of the NANOZR was observed by UV treatment. On the other hand, it was revealed that the C1s peak on the NANOZR surface was decreased by UV treatment (Figure 2). Analysis of the contact angle by using ultrapure water revealed that the surface of the UV treated NANOZR was superhydrophilic (Figure 3).

### 3.2. Evaluation of Protein Adsorption on the NANOZR Surface

**Figure 4:**
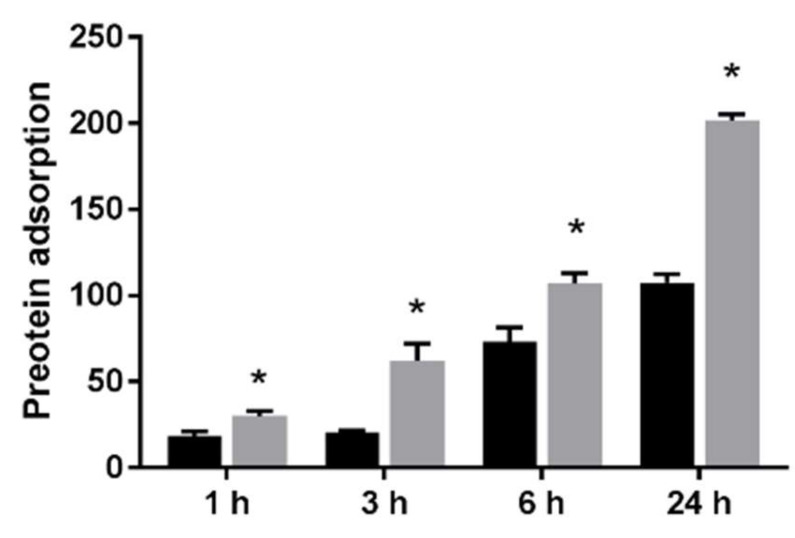
The effect of UV treatment on the surface of NANOZR on the adhesion of the material surface of BSA is shown. Evaluation time is 1 h, 3 h, 6 h and 24 h after incubation. BSA adhesion was significantly higher on the NANOZR surface of the test group compared to the control group at all counting times. (Gray bar—untreated surface, black bar—UV-treated surface). (*: *p* < 0.05)

The results of the adhesion examination of BSA on the UV-treated NANOZR and the untreated NANOZR surface are shown. BSA adhesion was significantly higher on the NANOZR surface of the test group compared to the control group at all counting times (Figure 4).

### 3.3. Effects of the NANOZR Surface on Cell Adhesion and Morphology in RBMCs, HUVECs, and RPLCs

**Figure 5:**
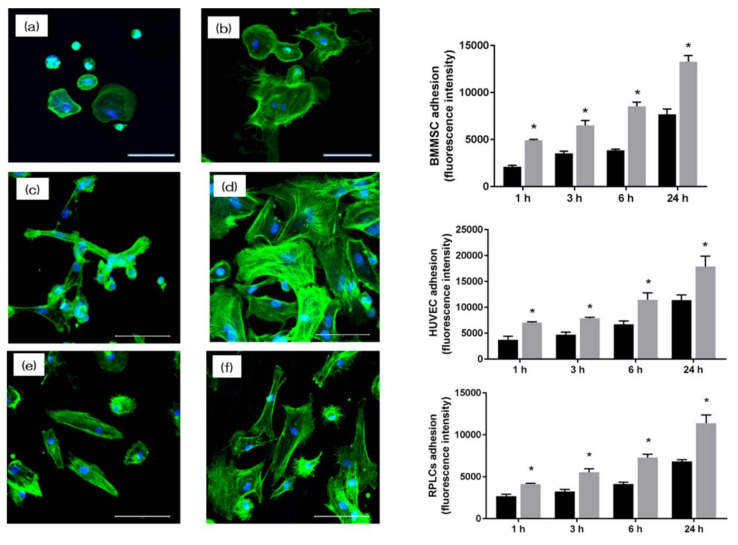
The morphology of rat bone marrow cells (RBMCs) (**a**,**b**), human umbilical vein endothelial cells (HUVECs) (**c**,**d**), and rat periodontal ligament cells (RPLCs) (**e**,**f**) on the surface of NANOZR after 24 h (RBMCs and RPLCs) and 6 h (HUVECs) of culture was observed with a fluorescence microscope (scale bar: 200 μm). At the same time, on the material surface of the experimental group, an increase in the number of cells and elongation of cell projections was observed as compared with the control group. In this experiment, the cell morphology was observed and the number of cells on the surface of each material was compared. At all measurement times, the adhesion number of RBMCs, HUVECs, and RPLCs in the test group was significantly higher than that in the control group. (Gray bar—untreated surface, black bar—UV-treated surface). (*: *p* < 0.05)

The morphology of RBMCs, HUVECs, and RPLCs on the surface of NANOZR after 24 h (RBMCs and RPLCs) and 6 h (HUVECs) of culture was observed with a fluorescence microscope. It was confirmed that various cells adhered to the surface of the materials of each group (Figure 5a–d). At the same time, on the material surface of the experimental group, an increase in the number of cells and elongation of cell projections was observed as compared with the control group. In this experiment, the cell morphology was observed and the number of cells on the surface of each material was compared. At all measurement times, the adhesion number of RBMCs, HUVECs, and RPLCs in the test group was significantly higher than that in the control group.

### 3.4. UV-Treatment Induced Bone Differentiation on the NANOZR Surface In Vitro

**Figure 6:**
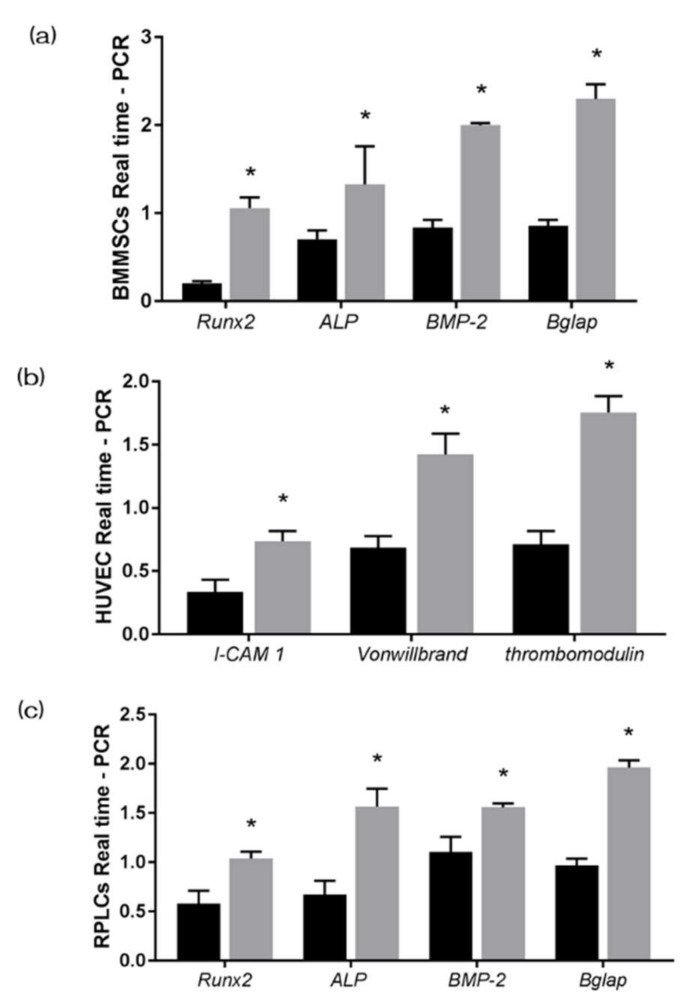
The gene expression related to the induction of hard tissue differentiation and the gene expression related to angiogenesis on the NANOZR surface of the test and the control group were analyzed. In this experiment, the assay was performed at a measurement time specific to each gene. Significantly higher gene expression was observed on the material surface of the test group at all measurement times. (Gray bar—untreated surface, black bar—UV-treated surface). (*: *p* < 0.05) (**a**) BMMSCs; (**b**) HUVECS; (**c**) RPLLCs.

**Figure 7:**
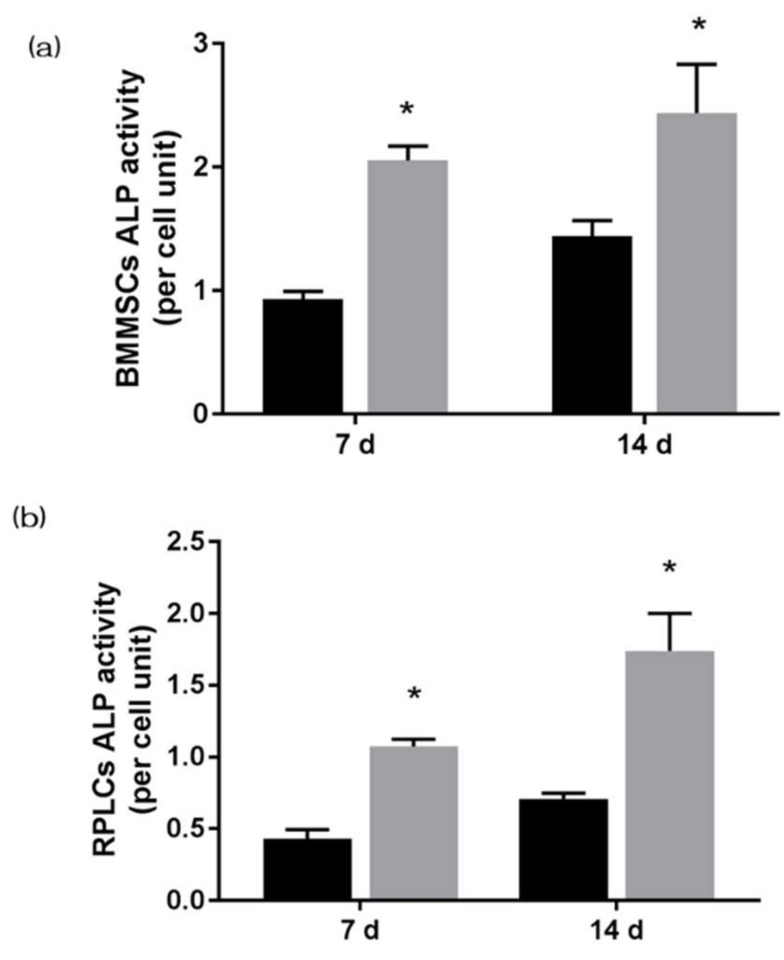
Alkaline phosphatase (ALP) expression was measured for bone differentiation, which is the initial reaction of induction of hard tissue differentiation. ALP expression in bone marrow cells seven and 14 days after the start of culture was significantly higher on the material surface of the test group. (Gray bar—untreated surface, black bar—UV-treated surface). (*: *p* < 0.05) (**a**) BMMSCs; (**b**) RPLCs.

**Figure 8:**
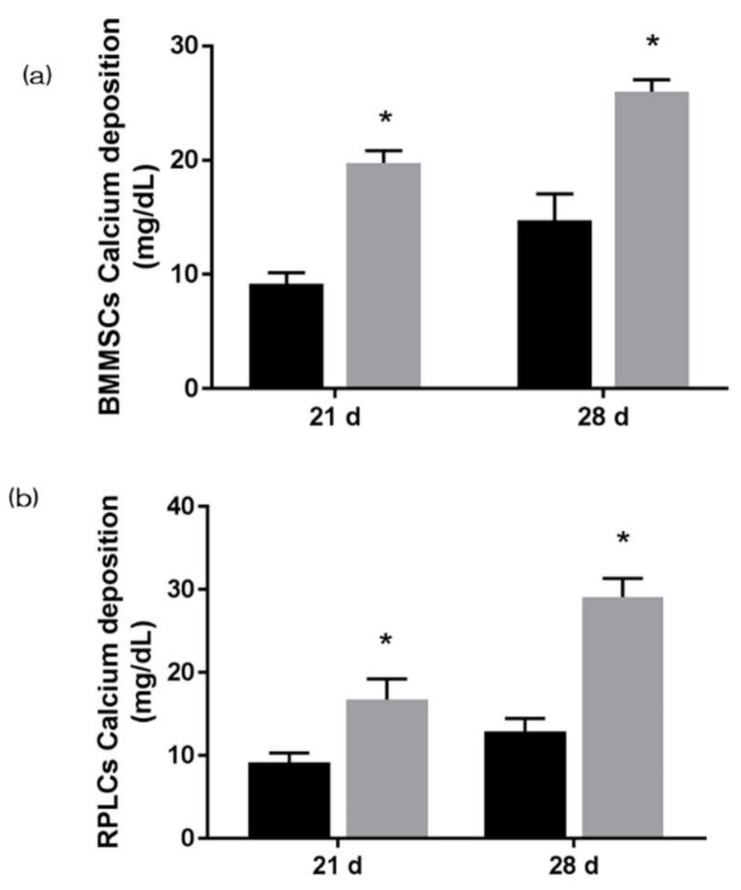
Mineralization was assayed for calcification, which is a late reaction of induction of hard tissue differentiation. The amount of calcium deposited 21 and 28 days after the incubation of culture was significantly high on the material surface of the test group. (Gray bar—untreated surface, black bar—UV treated surface). (*: *p* < 0.05) (**a**) BMMSCs; (**b**) RPLCs.

The gene expression related to the induction of hard tissue differentiation and the gene expression related to angiogenesis on the NANOZR surface of the test and the control group were analyzed. In this experiment, the assay was performed at a measurement time specific to each gene. Significantly higher gene expression was observed on the material surface of the test group at all measurement times (Figure 6). ALP expression was measured for bone differentiation, which is the initial reaction of induction of hard tissue differentiation. ALP expression in bone marrow cells seven and 14 days after the start of culture was significantly higher on the material surface of the test group (Figure 7). Mineralization was assayed for calcification, which is a late reaction of induction of hard tissue differentiation. The amount of calcium deposited 21 and 28 days after the incubation of culture was significantly high on the material surface of the test group (Figure 8).

### 3.5. Intracellular ROS Level and Mitochondrial Membrane Potential Change Detection for RBMCs

**Figure 9:**
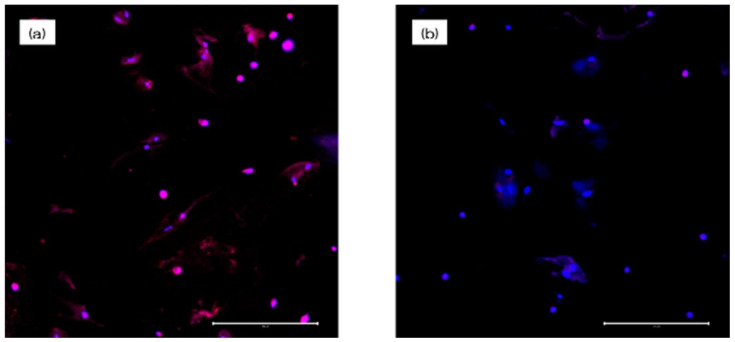
Macrophages on the NANOZR plate displayed a significantly higher level of intracellular ROS than those on the UV-NANOZR plate, along with the highest concentration of DNA. (**a**) untreated NANOZR; (**b**) UV-NANOZR. (scale bar: 200 μm)

Macrophages on the NANOZR plate displayed a significantly higher level of intracellular ROS than those on the UV-NANOZR plate, along with the highest concentration of DNA (Figure 9). These results indicated that the UV treatment may enhance the antioxidant properties of NANOZR.

**Figure 10:**
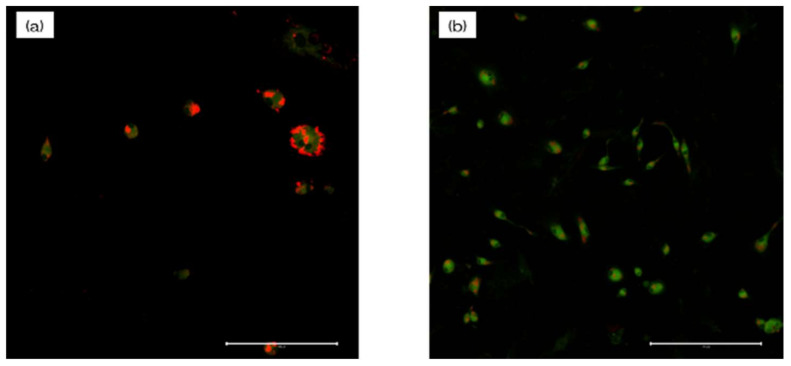
To measure mitochondrial function, mitochondrial membrane potential change was determined for the macrophages and was assessed by JC-1 staining. Fluorescence microscopy showed that in the test group, RBMCs displayed strong J-aggregation (red) and weak JC-1 monomer (green), while in the plate and Ti groups, RBMCs showed higher JC-1 monomer (green) levels with concomitantly decreased J-aggregation (red) due to low ΔΨm. This indicated that ΔΨm and oxidative stress on mitochondria were inhibited by UV-treated NANOZR. (**a**) untreated NANOZR; (**b**) UV-NANOZR. (scale bar: 200 μm)

To measure mitochondrial function, mitochondrial membrane potential change was determined for the macrophages and was assessed by JC-1 staining. Fluorescence microscopy (Figure 10) showed that in the test group, RBMCs displayed strong J-aggregation (red) and weak JC-1 monomer (green), while in the plate and Ti groups, RBMCs showed higher JC-1 monomer (green) levels with concomitantly decreased J-aggregation (red) due to low ΔΨm. This indicated that ΔΨm and oxidative stress on mitochondria were inhibited by UV-treated NANOZR.

## 4. Discussion

Various experimental reports have proved that a material surface modified by surface treatment has a higher hard-tissue-forming ability than the smooth material surface [3,4,5,6,7,8,9]. The surface treatment of inert NANOZR surface is therefore essential to achieve its potential for use in fabrication of implant materials. The effect of UV-treated NANOZR-based materials on biological properties, such as protein adsorption, attachment of cells (RBMCs, HUVECs, and HPLCs), angiogenesis, bone differentiation, and oxidative stress were examined in this study. No change was observed in the mechanical structure of the UV-treated NANOZR surface, but the amount of C on the material’s surface was reduced, and a superhydrophilic material surface was obtained. In the experiment using RBMCs and HPLCs, an increase in markers relating to the initial adhesion of RBMCs and HPLCs and the ability to induce hard tissue differentiation was observed for UV-treated NANOZR. In experiments using HUVECs, it was found that UV-treated NANOZR surfaces showed improved initial HUVECs and markers of gene expression related to angiogenesis. In experiments using HUVECs, improvement in the initial adhesion of HUVECs and the ability to heal wounds were observed for the UV-treated NANOZR surface. The UV-treated NANOZR surface showed low ROS and mitochondrial membrane potential (MMP) production and low oxidative stress, indicating less mitochondrial damage. Based on the abovementioned results, it was confirmed that UV treatment of the NANOZR surface induced a decrease in the carbon content and superhydrophilicity of the material surface and a reduction in oxidative stress. In addition, UV treatment showed beneficial effects on the behavior of various cells, and it was found that UV-treated NANOZR may be used as a novel implant material.

No difference in topography or roughness between nontreated and UV-treated NANOZR disks was observed using SEM and SPM. However, a decrease in the C peak and a rise in the O peak became clear for the UV-modified NANOZR surface by XPS analysis. The existence of carbon, which is a pollutant of implant material surfaces, is known to obstruct the adhesion of proteins and various cells participating in osseointegration [43,44,45,46,47,48,49,50,51,52,53,54,55,56,57,58,59,60,61,62,63] It is reported that UV treatment decreases carbon content on the titanium surface and the TZP material surface. Our observations agree with the results of these reports. The increase in the O peak on the surface of UV-treated NANOZR based on the XPS analysis is presumed to be due to the UV equipment used in this study. The device used is known to combine UV and ozone treatment. The result thus suggests that the material surface may be oxygen-rich. There are reports related to ozone treatment and cell activity, and the impact of this device on NANOZR increases its potential as an implant material [64,65]. It is said that the wettability of the material surface and the presence of carbon are closely related. The superhydrophilicity of the NANOZR surface due to the effect of UV treatment shown is considered to be correlated with the decrease of carbon by the results of this experiment. It is presumed that the material surface with less carbon and high wettability is effective for the initial adhesion of proteins related to induction of hard tissue differentiation [66]. In order for cells to adhere and grow on the material surface, adhesion of two types of proteins to the material surface is useful. These two types are adhesion-promoting proteins such as fibronectin and adhesion-inhibiting proteins such as albumin. It is believed that the induction of hard tissue differentiation is triggered only when two types of proteins coexist on the material surface. In other words, the results of this experiment indicate that UV treatment is useful as a device for improving the ability to induce hard tissue differentiation on the NANOZR surface. It is clear that the UV-treatment device used in this experiment made the NANOZR material surface superhydrophilic, which is consistent with this report. In this study, NANOZR surface was found to facilitate protein adsorption onto it.

To demonstrate the usefulness of implant materials with surface structure control as biomaterials, it is necessary to analyze the influence of a material surface on osseointegration, starting from wound healing [67,68]. Drilling causes bleeding and the formation of blood clots. Blood components release cell-adhesive extracellular matrix and cytokines involved in angiogenesis and bone formation [69,70]. In addition, HUVECs are known to have factors that cause RBMCs to migrate toward an implant. In this study, HUVECs were used to investigate the initial adhesion of vascular endothelial cells and angiogenesis on NANOZR surface [71,72]. In all experiments, high gene expression was observed on the UV-treated NANOZR surface. Therefore, UV-treated NANOZR is useful for early angiogenesis. Osteoblast function is essential for osseointegration. In general, there is a flow from RBMCs to initial adhesion, proliferation, differentiation, and calcification until osseointegration. In this study, we examined each of these processes. It was clarified that UV treatment on the NANOZR surface improves the initial adhesion of RBMCs. In addition, when the stained image of the RBMCs was confirmed, it was revealed that the elongation process of the cells was also very high because the wettability of NANOZR surface was high by UV treatment [73,74]. It was revealed that the UV-treated NANOZR surface enhances the expression of factors related to hard tissue formation in the tissue surrounding the implant placement. ALP activity, which is an early marker of induction of hard tissue differentiation, is an important factor in analyzing whether cells attached to the material surface change to hard tissue [75,76]. When changing from RBMCs to osteoblasts, these cells produce OCN and progress to calcification. The mineralization and Bglap mRNA expression evaluated in this experiment are considered as late markers for induction of hard tissue differentiation. It is believed that UV-treated NANOZR accelerates the flow leading to a series of induction of hard tissue differentiation and induces early bone formation [60]. It is considered that the elongation of cell processes and the strong adsorption of proteins are due to enhanced differentiation and calcification. The presence of periodontal tissue is important to ensure that the initial fixation after implant placement is maintained. Periodontal ligament cells are useful for periodontal tissue regeneration [77,78]. Regeneration of periodontal tissue around an implant requires the formation of periodontal-like tissue and cementum-like hard tissue due to the proliferation of periodontal ligament cells [79,80]. For this evaluation method, we used amelogenin-coated nanostructured pure titanium metal. In this study, we revealed that the UV-treated NANOZR surface is useful for improving periodontal ligament cell proliferation and hard tissue differentiation induction. Another study showed that a rough titanium surface improved periodontal ligament cell and osteogenic differentiation [35,36]. To avoid the interference of connective tissues, an implant surface should be engineered to promote osteogenic differentiation and a faster healing process. In a previous study investigating several roughed implant surfaces, we found that nanomodified material surface can enhance the adhesion of endothelial cells and the expression of genes encoding angiogenic factors and adhesion molecules [35]. Experiments using three types of cells revealed that the UV-treated NANOZR surface is useful for angiogenesis, hard tissue differentiation induction and calcification, and periodontal tissue regeneration.

UV treatment is known to directly decompose oxygen and generate oxygen radicals, and the higher the light energy, the better the ability to decompose organic substances [81,82,83]. Further, it was found that active oxygen generated at the time of UV irradiation can react with an organic compound to form a new functional group on the material surface to increase its hydrophilicity and modify it. Since implant treatment always involves a surgical procedure, oxidative stress due to inflammation often occurs around the implant. It is known that excessive amounts cause apoptosis of cells and delay of tissue healing, that is, failure of implant placement. In other words, suppression of ROS generation is important for successful implant treatment. UV treatment suppressing the generation of oxidative stress by producing active oxygen was shown in some studies [81,82,83]. In this study, evaluation of ROS and mitochondrial membrane potential using bone marrow cells revealed that UV treatment on the NANOZR surface suppressed the occurrence of oxidative stress on the material surface. In another report, reduction of C on the surface of the material is shown to be caused by suppression of ROS generation [81,82,83]. Therefore, UV treatment may make NANOZR a biofunctional material. In the future, the usefulness of this material should be examined by bacterial experiments and in vivo tests. If the idea of this experiment succeeds, it will be possible to apply it clinically in two ways. The first is the realization of implant treatment for patients with metal allergies. The second is the early formation of hard tissue after implant placement. There are various obstacles to the realization, but we will continue to aim at the realization of research close to clinical practice.

## 5. Conclusions

In this study, we found that UV treatment of the surface of the NANOZR material removed carbon on the material surface and increased surface hydrophilicity. In addition, the in vitro study revealed that the formation of reactive oxygen species on the material surface and suppression of the occurrence of oxidative stress are useful for improving angiogenesis, the ability to induce hard tissue differentiation, and the regeneration of periodontal tissue.
